# The Rover Environmental Monitoring Station Ground Temperature Sensor: A Pyrometer for Measuring Ground Temperature on Mars

**DOI:** 10.3390/s101009211

**Published:** 2010-10-15

**Authors:** Eduardo Sebastián, Carlos Armiens, Javier Gómez-Elvira, María P. Zorzano, Jesus Martinez-Frias, Blanca Esteban, Miguel Ramos

**Affiliations:** 1 Centro de Astrobiología (CSIC-INTA), Ctra. Ajalvir Km. 4, 28850 Torrejón de Ardoz, Madrid, Spain; E-Mails: armiensac@inta.es (C.A.); gomezej@inta.es (J.G.-E); zorzanomm@inta.es (M.P.Z.); martinezfj@inta.es (J.M.-F.);; 2 Department of Physics, University of Alcalá, Ctra. N-II Km33. Alcalá de Henares, Spain; E-Mails: blanca.esteban@uah.es (B.E.); miguel.ramos@uah.es (M.R.)

**Keywords:** IR ground temperature sensor, sensor thermal model, spacecraft instrumentation, in-flight calibration

## Abstract

We describe the parameters that drive the design and modeling of the Rover Environmental Monitoring Station (REMS) Ground Temperature Sensor (GTS), an instrument aboard NASA’s Mars Science Laboratory, and report preliminary test results. REMS GTS is a lightweight, low-power, and low cost pyrometer for measuring the Martian surface kinematic temperature. The sensor’s main feature is its innovative design, based on a simple mechanical structure with no moving parts. It includes an in-flight calibration system that permits sensor recalibration when sensor sensitivity has been degraded by deposition of dust over the optics. This paper provides the first results of a GTS engineering model working in a Martian-like, extreme environment.

## 1. Introduction

NASA’s Mars Science Laboratory (MSL) is the first mission to include an environmental station, the Rover Environmental Monitoring Station (REMS), located on the Rover and with a mission duration of one Martian year to enable a study of Martian seasons [1,2]. Its launch is scheduled for the fall of 2011. REMS has been developed by the Spanish Centro de Astrobiología (CSIC-INTA) in collaboration with EADS-Crisa, the Universidad Politécnica de Cataluña, the Finnish Meteorological Institute, the NASA Ames Research Centre, the University of Michigan, the Universidad de Alcalá and the California Institute of Technology. REMS has been designed for measuring ambient pressure, humidity, wind speed and direction, UV radiation, and air and ground temperature [3]. Specifically, the Ground Temperature Sensor (GTS) is a pyrometer designed to measure the kinematic temperature of the Martian surface.

As a result of previous NASA missions, it is well known that the average planet surface temperature on Mars is 220 K and varies widely over the course of a Martian day, from 145 K during the polar night to 300 K on the equator at midday at the closest point in its orbit around the Sun, with diurnal variations of up to 80–100 K. Near-surface atmospheric temperatures at potential landing sites (e.g., Gusev crater, Meridiani Planum) range from 173 K to 273 K. Much more recent measurements taken by Phoenix (25 May 2008) indicate that Martian regolith temperatures (polar latitudes) range from 181 K to 253 K. Additionally, Mars undergoes very extreme ground temperature gradients between the ground and the atmosphere at 1.5 m above the surface, with differences of ±40 K [4].

This huge variation in diurnal temperature has a dramatic effect on static stability and hence on the dynamics of the Martian planetary boundary layer. The thermal structure and dynamics of the atmosphere are strongly influenced by the exchange of moisture, heat, mass, and momentum between the surface and atmosphere. However, the causes of many significant temperature variations still remained unexplained. For instance, when Martian surface temperatures and albedos were measured using ground-based IR spectroscopy, between September and December 1988 [5], these measurements indicated surface temperatures which seemed to be around 30 K higher than the Viking temperatures measured in 1977, and closer to the theoretical temperatures calculated from the Viking Primary Mission in 1976.

Retrieval of the *in-situ* surface temperature of Mars is essential to develop environmental models of the Martian atmosphere-surface boundary layer [6,7]. Stability, surface heat fluxes, and growth of the atmosphere-surface mixed layer can be estimated from ground and atmospheric temperatures [8]. An important consideration related to the temperature of the Martian surface environment is that it can be influenced by different factors (among others, putative radioactive heat sources, mantle heat flow, surface temperature, thermal conductivity and, particularly, the mineralogy of the Martian regolith) [9].

From a technical point of view, *in-situ* Martian surface ground kinematic temperature measurements can primarily be performed in two different ways. The first is the use of contact sensors located a few millimeters below the surface, for example, the NetLander ATMIS instrument [8] or the TECP probe contained in the Phoenix MECA instrument [10]. Despite their simplicity, these kinds of measurements are not always possible due to mission restrictions, and this is the case for the REMS on MSL. Practical problems include the thermal influence of the probe when deploying the transducer into the ground, and the existence of a thin layer of dust over the rocky surface of Mars, which could generate temperature gradients between the surface and the first few millimeters of subsurface, disturbing the measurement. One alternative would be the use of contactless sensors, using IR spectrometers and radiometers as pyrometers. An example of an IR spectrometer which has taken Martian atmosphere and ground brightness temperature measurements is the Mini-TES on the NASA Spirit and Opportunity Rovers [4]. Nevertheless, the clearest example of a pyrometer used for the *in-situ* determination of surface temperature in a space application is the Multipurpose Sensors for Surface and Sub-Surface Science-Thermal Probe (MUPUS-TP) experiment on the ROSETTA mission [11].

In general, the measurement of temperature using IR techniques is more complex than using contact sensors due to the existence of problems associated with the physical measurement procedure. This can also be applied to the measurement of Martian surface kinematic temperature using a pyrometer. The uncertainty in ground emissivity (*ε*) is perhaps the most important difficulty, resulting in different kinematic and brightness temperatures. Typical emissivity values of Martian soils from 6 to 25 μm vary between 0.9 and 1 [12], introducing significant uncertainty into the power emitted and reflected by the ground. Thus, in order to achieve high surface temperature accuracy, the value of soil emissivity must be estimated or measured. This explains the need for specific studies of the IR reflectance properties associated with different kinds of Martian surface material such as minerals and rocks. Recently, the FTIR (Fourier Transform Infrared Spectroscopy) reflectance of a set of selected astrobiologically significant minerals (including oxides, oxi-hydroxides, sulfates, chlorides, opal and clays) and basalt (as the main and most widespread volcanic Martian rock) was measured, considering different mixing amounts, and covering the specific working wavelength range of the REMS GTS [13]. The results obtained indicated significant percentage increases or decreases in reflectance over the entire wavelength range (e.g., basalt-hematite *vs*. basalt-magnetite), and specific variations restricted to some spectral bands (e.g., basalt-smectite *vs*. basalt-opal). Another alternative is the use of color pyrometry techniques [14,15] to estimate the emissivity value. Additionally, since emissivity is different to 1 and the surface reflectivity is assumed to be *r =* 1 *− ε*, IR solar radiation, as well as IR energy coming from the environment, mainly the Rover or lander and the atmosphere, augment ground emissions. Again, these factors must be taken into account and compensated if precise temperature estimation is required.

Another factor that may disturb ground temperature determination is atmospheric absorption. The Martian atmosphere consists mostly of CO_2_, which has a strongly absorbing band centered at 15 μm. The CO_2_ in the atmospheric column within the sensor view cone may also act as absorber and emitter, since the air is generally at a very different temperature from the ground. Also, water molecules have a very strong absorption at 1.45 μm, and a weak absorption at 6.27 μm [16]. These atmospheric absorption bands must be considered in order to define the pyrometer spectral response.

Finally, there is a further factor which could affect measurement accuracy: a dusty atmosphere. First because dust absorbs and emits IR energy inside the measurement band, disturbing the energy coming from the ground, and second, because dust could cause a deterioration in pyrometer optical performance. It could be deposited over the detector, blocking the IR radiation emitted by the ground. Therefore, systems for recalibrating pyrometer sensitivity or avoiding dust deposition must be included in Martian pyrometers.

Table 1 summarizes the main advantages and drawbacks of the principal techniques used to measure ground temperature.

## 2. REMS GTS Description

The REMS GTS is a lightweight, low-power, and low cost pyrometer for measuring the Martian surface kinematic temperature. The GTS works by integrating the IR energy radiated by the ground, with a temperature range between 150 K and 300 K. Derived from REMS scientific requirements [3], the GTS aims to achieve an accuracy of ±5 K and resolution of 0.1 K (Table 2).

**Figure 1.**
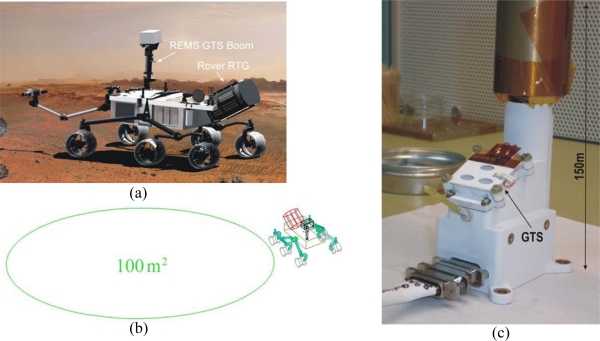
(a) Recreation of the NASA MSL *Curiosity* Rover on Mars; (b) Layout of REMS GTS focused ground area; (c) A photograph of REMS Boom 1 Flight Model.

The GTS is located in one of the REMS booms (Figure 1c), positioned in the NASA/MSL Rover mast at 1.6 m height (Figure 1a). The boom is shaped like a small arm, 150 mm long, and hosts the electronics employed to amplify thermopile signals. To avoid local temperature effects, the GTS focuses on a large ellipsoidal ground surface area of around 100 m^2^, measuring its average temperature (Figure 1b). The field of view (FOV) and its orientation was selected to avoid Rover direct vision, but the area is not far enough from the Rover to rule out its influence. The MSL Rover Radioisotope Thermoelectric Generator (RTG) can reach temperatures of 200 K above atmospheric temperature, and a simplified thermal model has been developed to quantify and bound the ground temperature uncertainty that it generates. For this study, the influence of two effects, ground reflections and ground warming up, were analyzed. Ground temperature uncertainty was bounded, taking a maximum value of +3.75 K. The uncertainty may be partially compensated on Mars by using the same mathematical models as those employed for the analysis and the real, *in-situ* RTG temperatures. In the following subsections a description is given of the specific characteristics of the GTS design.

### 2.1. Mechanical Design

The GTS mechanical structure is an *ad-hoc* design which tries to provide the best working conditions given the restricted resources available to the REMS. From a technical point of view, one of the most important sources of errors in radiometers and pyrometers is the existence of spatial and temporal thermal gradients in the housing of the IR detectors, restricting sensor sensitivity. Generally, one of the methods employed to minimize their influence is the use of a thermal inertial mass around the sensor to reduce the gradients. Nevertheless, there are some applications in which this solution may not be sufficient, for example, open air applications, due to the effect of the sun and wind, or applications where the focused surface is so hot that it heats the entire pyrometer structure. Research is still underway to find a solution to this problem [17]. In [18], the authors proposed a model based on a simplification of thermopile internal thermal behavior, using variations in the thermopile internal temperature sensor to compensate the effect of the gradient. In [19], a new design was described that included two transducers with different levels of sensitivity to incoming IR radiation in order to compensate spatial gradients between the front and bottom sections of an IR detector. Thus, from the output signal of both transducers, a system of two equations can be established from which the equations for incoming radiation can be solved avoiding the influence of the gradients. Nevertheless, the most widely used means of dealing with sensor temperature instabilities and gradients is the use of mechanical choppers [12,20]. These choppers also prevent errors due to electronics bias drift, as well as compensating degradation of the sensor due to changes in its sensitivity, optics properties, and thermal gradients. In contrast, these systems necessitate the use of mechanical actuators and mirrors, reducing instrument robustness and increasing energy demands and the risk of breakage.

**Figure 2.**
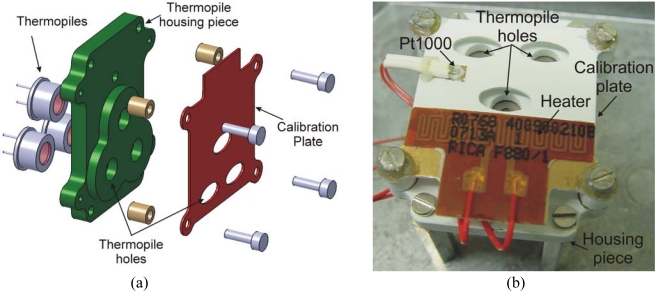
(a) The 3D mechanical layout of REMS GTS; (b) A photograph of the REMS GTS flight model.

The GTS design must address these general problems, but it must also consider available REMS resources, and restrictions such as the lack of a temperature stabilization control system and the impossibility of using mechanical choppers due to robustness and power consumption issues. Thus, the mechanical structure of the sensor is composed of a metal housing piece containing the IR detectors, and which further functions as thermal mass to ensure acceptably low drift in detector case temperature. Furthermore, an in-flight recalibration system is required, due both to the dusty atmosphere on Mars and the long mission duration [1,2], which may imply the deposition of dust on the detectors’ optics, leading to a deterioration in their output signals. This system is implemented by a simple low mass and high emissivity calibration plate (Figure 2), which can be heated to a given temperature and is placed in front of the thermopile housing piece, so that each IR detector looks at the ground through a hole in the plate. Thus, part of the FOV is obstructed by the calibration system, limiting the measurement solid angle (Table 2). The plate temperature is measured using a specific Resistor Temperature Detector (RTD), a Pt1000, attached to its surface. To our knowledge, this is an innovative and pioneering pyrometer recalibration system with no moving parts, designed to compensate deterioration of the sensor in a dusty environment, whilst avoiding complicated and costly commercial air purge systems to maintain the sensor window free of dust. There is no alternative to the proposed recalibration system for REMS.

### 2.2. Infrared Detectors and Temperature Sensors

In pyrometer design, two different kinds of IR detector are generally available: photonic and thermal detectors. The main advantages of photonic detectors (photoconductors, photodiodes and phototransistors) are their high detectivity and speed. Nevertheless, they are very sensitive to any change in their temperature, such changes leading to drastic variations in detectivity and occasioning long term drift. Additionally, they are only sensitive to restrictive wavelength channels. On the other hand, the temperature of thermal detectors (bolometers, pyroelectric sensors and thermopiles) is more stable and they are sensitive to the entire wavelength spectra, but present lower detectivity.

In the case of REMS, and given the available resources, there is virtually no alternative to thermopiles. The reasons for this are multiple: these detectors have the advantage of being capable of functioning at almost any operational temperature, and they are small and lightweight since modern semiconductor technology has made it possible to produce thermopile sensors consisting of hundreds of thermocouples within an area of several square millimeters. In addition, they are sensitive to all the IR spectra, comparatively cheap and require simple readout electronics, enabling further reductions to be made in the weight and size of the complete instrument. Finally, thermopiles can operate without the need for any sort of temperature control system, because they are less sensitive than other systems to the emergence of thermal gradients. This aspect has been analyzed in [21], where the author proposed an internal thermal equilibrium equation for unchopped IR detectors in order to model sensitivity to changes in instrument temperature. The study was carried out for two different kinds of thermal detectors, a thermopile and a bolometer, and showed that the thermopile outperformed the bolometer since it obtains an output based on differential temperature, which is less affected by changes in sensor temperature.

However, thermopiles are not standard parts for space or military applications and at present no formally space-qualified thermopile sensors exist. Nevertheless, it should be noted here that the Infrared Thermal Mapper (IRTM) experiment on the VIKING mission [12] and the MUPUS-TP experiment on the ROSETTA mission [11] have proven the suitability of this kind of detector for measuring low object temperatures under space conditions.

**Figure 3.**
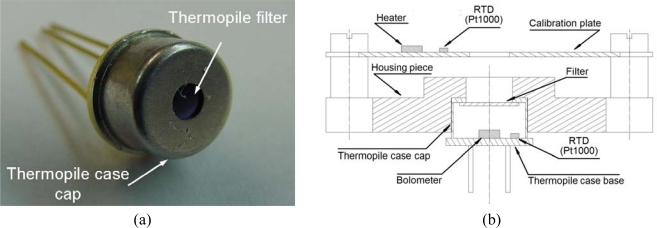
(a) A photograph of the IPHT TS-100 thermopile; (b) The 2D mechanical layout of REMS GTS and a thermopile.

The thermopile model selected for the REMS is the TS-100, from the Institute for Physical High Technology (IPHT) in Jena (Germany), encapsulated within a TO-5 with no optical system but rather a thermopile filter built to specifications and pre-bonded onto the TO-5 as the thermopile window (Figure 3a). The thermopiles have a non corrosive insulator and transparent atmosphere filling, as well as an internal RTD, a Pt1000, to measure temperature at the thermopile case base, which acts as a temperature reference for the thermocouple cold-junction (Figure 3b). The Pt1000s used consist of thin film platinum thermo-resistors embedded into an alumina substrate; more specifically, they comprise the P1k0.161.7W.A.010 Class A from the company Innovative Sensor Technology (Minisens). Thermo-resistors similar to these have been in use on previous space missions on Mars since the Mars-96 landers.

The GTS uses three different thermopiles on three different infrared wavelength channels, A, B and C (Table 3). The first two bands are optimized for the upper and lower Martian ground temperature ranges. Following Wien's law, the maximum blackbody spectral radiance for a given temperature is given by *λ_max_*[μm] = 2,898/*T* [K]. If the maximal and minimal Martian temperatures are 300 K and 150 K, then the sensor is designed to work optimally in the range from 9.9 μm to 19.3 μm.

Additionally, the measurements must be performed within a range where the ratio of IR radiance emitted by the Martian surface to the solar IR radiance reflected by the Martian surface, for typical Martian soil emissivities, is significantly greater than one. This condition is achieved above 8 μm, where the solar reflected radiance is smaller than 0.5% for the lower ground temperature. Finally, the last restriction for the selection of these bands is avoidance of the CO_2_ atmospheric absorption band centered at 15 μm and bandwidth of 1 μm, the main component of the Martian atmosphere. The readout signals of these two thermopiles can be combined in order to apply color pyrometry techniques [15], and in certain specific circumstances, this can help to estimate Martian ground emissivity. The third band is centered on the CO_2_ absorption band. This allows any residual influence that the atmosphere may have on the other two thermopile bands to be determined.

Finally, the calibration plate is heated using an electrical heater consisting of an etched-foil resistive heating element laminated between layers of polyimide, a flexible and thin insulator. This kind of heater is robust, accurate, reliable and ideal for applications with space and weight limitations, or where the heater will be exposed to a vacuum.

### 2.3. Electrical Design and Data Collection Modes

**Figure 4.**
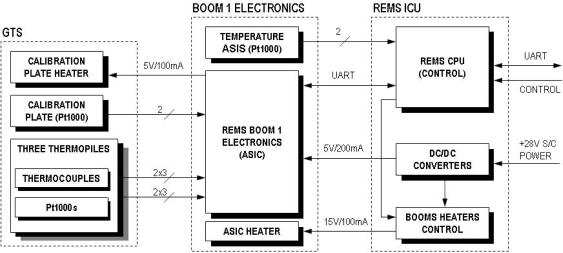
REMS GTS electronics architecture.

The GTS electrical output signals (thermopile output voltage and the resistance of the different Pt1000s, those inside the thermopiles and the one on the calibration plate) are sampled by REMS electronics. This electronic system presents a distributed architecture (Figure 4).

In general terms, the Instrument Control Unit (ICU) is located inside the MSL Rover body, whereas the data acquisition electronics are located inside REMS booms [3]. This architecture permits optimization of the total harness, and at the same time reduces coupled noise in analog signals. Nevertheless, a heater is required to warm up the electronics located in the boom for temperatures below the operational range, since the boom remains at atmospheric temperatures.

**Figure 5.**
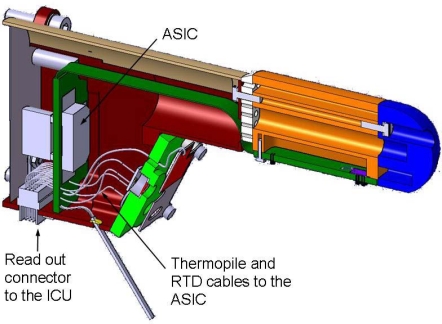
A 3D electro-mechanical model of REMS GTS and Boom ASIC.

**Figure 6.**
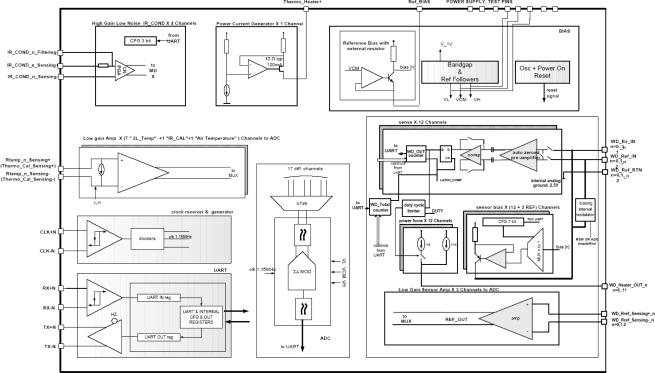
Simplified ASIC block diagram. Thermopile amplifier (High Gain Low Noise IR_COND), Pt1000 amplifiers (Low gain Amp), calibration plate heater driver (Power Current Generator).

REMS data acquisition requires the use of a mixed signal (Digital-Analog) Application Specific Integrated Circuit (ASIC) to implement the REMS sensor and driver front-end interface, due to space and weight restrictions. The ASIC is located in the rear part of the boom (Figure 5), and has been designed ad-hoc for this application using radiation tolerant technology, XFAB 1 μm CMOS SOI. The objective of the REMS ASIC is to condition REMS sensor signals and send sensor data to the ICU on ICU demand. It provides the analog circuitry needed for the amplification and conditioning of sensors and drivers, as well as an integrated 16 bits analog-to-digital converter to convert analog signals into digital data. There is also a digital section comprising the control unit, which controls the entire acquisition process from the sensors, and store the values into a bank of registers and an UART (Figure 6). The REMS ASIC communicates with the ICU using a dedicated point to point serial RS-422 interface. Communication between the ICU and the Rover CPU is also implemented using the same bus.

The thermopile amplifiers (IR_COND) (Figure 6) are fully differential to reduce sensor and amplifier common-mode noises. It is important to note that the thermopile signal is bipolar and floating, with no external voltage reference, and it is referenced internally. Additionally, to achieve the low offset required, a chopped technology based on a switch capacitor stage, which applies correlate double sampling methodology operating at 4.5 KHz, is used. The amplifier gain is programmable (PGA) from 64 to 2,048 to accommodate the different thermopile output ranges, which can change depending on the temperature difference between the thermopiles and the ground, and the type of thermopile filter used. Finally, the channel band width is limited using an external feedback capacitor.

The Pt1000 channels drain a constant 1mA current through the sensor resistor, and the voltage drop is amplified and measured. It is important to note that this current is only active during ADC measurement of the RTDs, 1/16 of second, to avoid self heating of the sensor. This will be performed automatically by the ASIC control unit. Finally, the calibration plate heater is driven using a 100 mA electric signal which allows us to attain temperatures about 14 K above the temperature of the surrounding atmosphere. ASIC channel calibration is performed to compensate amplifier parameter deviation. Table 4 summarizes the most relevant characteristics.

The software used to manage GTS behavior is executed in the REMS ICU inside the CPU, where the Boom1 ASIC acts as a peripheral of the CPU. This software establishes two GTS operational modes. The first is the normal mode, which carries out a systematic sampling of all REMS and GTS variables. The sampling lasts 5 min and it is executed every hour, using a sampling period of 1 s. The second mode is specific to the GTS and is used to run the in-flight calibration algorithm. It consists of an extended sampling period of 15 min, in which the calibration plate heater is switched on for the last 14 min, whilst the sampling period remains constant at 1 s.

In both operational modes, REMS software executes a function to carry out the automatic gain setting of the thermopile channels before starting the sampling. The objective is to configure the maximum gain without saturating the amplifiers. In general terms, the function works by configuring the highest gain and taking a sample; if the amplifier output module is below a saturation security bound, that gain is set, and if not, the selected gain is reduced a level and the procedure is executed until the correct gain is found.

Table 5 summarizes the GTS data production and power budget derived from the operational modes described in the previous paragraph, considering only the energy directly consumed by the GTS sensors and heater. Two considerations have been assumed: first, that the in-flight calibration algorithm is executed once per week, and second, that ASIC power consumption and ASIC energy necessary to reach operational temperature is not considered.

## 3. REMS GTS Model

The REMS GTS has been modeled using an innovative set of equations, because they consider explicitly the sensor’s internal and external physical structure and operation. The proposed model is based on an energy balance equation that accounts for the heat fluxes exchanged by radiation, conduction and convection between thermopile detector and the elements around. Despite being more mathematically complex than that commonly used [22], this model has permitted the design of practical methodologies to compensate the effects of sensor spatial thermal gradients, and to calibrate model constants based on a differential approach.

**Figure 7.**
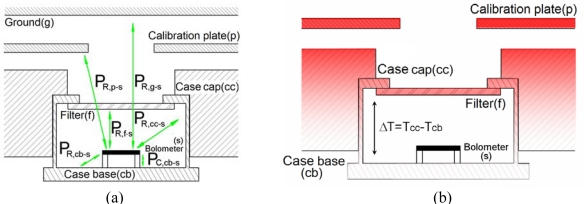
(a) Simplified diagram of GTS heat fluxes; (b) GTS and thermopile thermal gradient.

The model starts from the definition of an energy balance equation Equation (1) which accounts for the heat fluxes entering the thermopile bolometer from all the bodies around it, disregarding the heat fluxes between physical elements other than the bolometer (Figure 7a). The bolometer is designed to be well insulated from the thermopile case and to have low thermal mass, so that the equilibrium condition is reached after a setting time of a few milliseconds.

(1) $$P_{R,g-s}+P_{R,p-s}+P_{R,f-s}+P_{R,cc-s}+P_{R,cb-s}+P_{C,cb-s}=0$$

The terms *P_R,x−x_* and *P_C,x−x_* of Equation (1) represent the heat power exchanged by radiation, and conduction and convection, respectively, whilst the subscripts *x* refer to the bodies that exchange the heat (*g* is for the ground, *p* for the calibration plate, *f* for the filter, *cc* for the thermopile case cap, *cb* for the thermopile case base and *s* for the bolometer). Based on simplified one dimensional heat transfer models [23,24], Equation (1) can be expressed in terms of Equation (2), assuming two simplifications:

1. The temperature of the atmosphere inside the thermopile is equal to the temperature of the case base.
2. The bolometer filter FOV, which is limited by the shape of the thermopile case, is equal to the sum of the bolometer FOV of the ground and the calibration plate FOV.

(2) $$\alpha \cdot K_{1}\cdot \Phi_{g}+\left(1-\alpha \right)\cdot K_{1}\cdot \Phi_{p}+K_{1}\cdot \Phi_{f}+\frac{K_{2}}{2}\cdot \Phi_{\mathrm{cc}}+\frac{K_{2}}{2}\cdot \Phi_{\mathrm{cb}}-\left(K_{1}+K_{2}\right)\cdot \Phi_{s}+K_{3}\cdot \left(T_{\mathrm{cb}}-T_{s}\right)=0$$

The constant *α* represents the factor of the thermopile FOV unobstructed by the flight calibration plate, whilst constants *K_1_*, *K_2_* and *K_3_* group a set of physical constants such as areas, volumes, view factors, conductivities and convection coefficients. All of them are subject to calibration.

Meanwhile, the heat flux terms Φ*_x_* are calculated using Planck’s law, where *x* refers to the body. These terms depend on the transmittance of the thermopile filter, bolometer absorbance, and the temperatures and emissivities of the different bodies, such as the thermopile case base (*T_cb_*) and calibration plate (*T_p_*), which can be directly measured using the specific Pt1000 temperature sensors, the thermopile bolometer (*T_s_*) and thermopile case cap (*T_cc_*), which are measured indirectly as will be described below, and thermopile filter (*T_f_*), which is assumed to be equal to the temperature of the thermopile case cap since they are in good thermal contact. Note that all the flux terms are known except for Φ*_g_*, which is the unknown in the equation, and through its determination, the kinematic temperature of ground soil can be calculated.

The temperature of the bolometer, *T_s_*, is obtained from the output voltage of the thermopile. The thermopile produces a voltage representation of the temperature difference between its case base (cold-junction) and the bolometer (hot-junction), Equation (3). The GTS thermopiles have 100 thermocouples connected in series and embedded between the case base and the bolometer, and the term *α_AB_*|_*T_cb_*_ stands for the Seebeck coefficient related to the association of the two thermopile thermocouple materials.

(3) $$V_{\mathrm{out}}=100\cdot \alpha_{\mathrm{AB}}{|}_{T_{c}}\cdot \left(T_{s}-T_{cb}\right)$$

Finally, the temperature of the thermopile case cap, *T_cc_*, is essentially the same as the temperature of the base, *T_cb_*, since both are in good thermal contact. Nevertheless, the calibration plate, as is shown in Figure 2, is screwed to the thermopile housing piece, creating a conduction thermal coupling between these two pieces and by extension with the thermopiles. In this way, small thermal gradients appear between the thermopile case cap, the top part, and the base or the bottom part, during the heating of the calibration plate. From this fact, a relationship between the overall temperature of the calibration plate and the temperature difference between the thermopile case base and case cap can be established Equation (4). This relationship is assumed to be lineal throughout the temperature-independent constant, *K_p−c_*, which is also calibrated:

(4) $$T_{\mathrm{cc}}-T_{\mathrm{cb}}=K_{p-c}\cdot \Delta T_{p}$$

### 3.1. In-Flight Calibration Equations

One of the main GTS priorities is how to resolve the gradual build-up of dust on the filters. Whilst operating under Martian conditions and during the landing process, dust may collect on the thermopile filter. Dust has high emissivity and can block light into and out of the detector, and when in contact with the filter acquires the same temperature. Thus, dust can be seen as changing the area of the filter into something similar to the case. In other words, if we define a factor *β* representing that part of the FOV which has not been obstructed by dust, Equation (2) can be rewritten as follows:

(5) $$\beta \cdot \alpha \cdot K_{1}\cdot \Phi_{g}+\beta \cdot (1-\alpha )\cdot K_{1}\cdot \Phi_{p}+\beta \cdot K_{1}\cdot \Phi_{f}+\frac{K_{2}}{2}\cdot \Phi_{cc}+\frac{K_{2}}{2}\cdot \Phi_{cb}-\left(\beta \cdot K_{1}+K_{2}\right)\cdot \Phi_{s}+K_{3}\cdot \left(T_{cb}-T_{s}\right)=0$$

Therefore, factor *β* must be determined during operations. This can be done by varying the temperature of the calibration plate through heating it up, whilst ground temperature remains stable. Thus, using the Equation (5) for two different calibration plate temperatures, the system of Equation (6) can be defined:

(6.1) $$\beta \cdot \left\lfloor \alpha \cdot K_{1}\cdot \Phi_{g}+(1-\alpha )\cdot K_{1}\cdot \Phi_{p1}+c_{1}\right\rfloor +d_{1}=0$$

(6.2) $$\beta \cdot \left\lfloor \alpha \cdot K_{1}\cdot \Phi_{g}+(1-\alpha )\cdot K_{1}\cdot \Phi_{p2}+c_{2}\right\rfloor +d_{2}=0$$

where *c*_1_ = *K*_1_·Φ*_cc_*_1_ − *K*_1_·Φ*_s_*_1_, *c*_2_ = *K*_1_·Φ*_cc_*_2_ − *K*_1_·Φ*_s_*_2_, d1=K22(Φcc1+Φcb1)−K2⋅Φs1+K3⋅(Tcb1−Ts1), d2=K22(Φcc2+Φcb2)−K2⋅Φs2+K3⋅(Tcb2−Ts2) are a set of known heat terms, in which it is assumed that *T_cc_*, *T_cb_* and *T_s_* may be different for each of the two calibration plate temperatures. Finally, the system Equation (6) can be solved for the factor *β*, eliminating the unknown but constant term *α·K*_1_·Φ*_g_* and depending only on measured temperatures and calibrated constants:

(7) $$\beta =\frac{d_{2}-d_{1}}{\left(1-\alpha \right)\cdot K_{1}\cdot \left(\Phi_{p1}-\Phi_{p2}\right)+c_{1}-c_{2}}$$

## 4. Field Test Campaign

**Figure 8.**
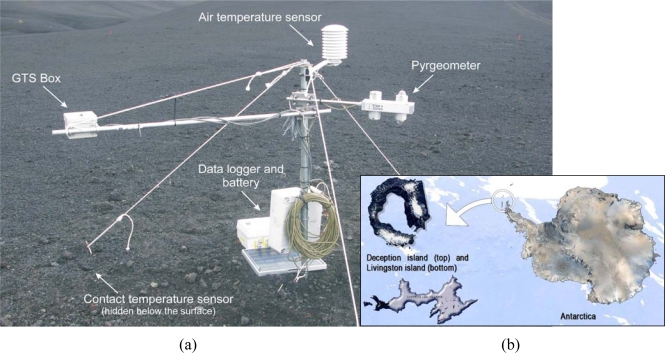
(a) Deployment of the REMS GTS: field test at Antarctica; (b) Location of the Spanish Antarctic Station on Deception Island.

A field test campaign was designed to check REMS GTS performance, validating the proposed sensor model and its previous calibration. In January 2009, an engineering model of the GTS was deployed in Antarctica in the surroundings of the Spanish Antarctic Station on Deception Island (an active volcano located in South Shetland archipelago, Maritime Antarctica; see Figure 8b). This site was chosen as it represents a remote and hostile environment with harsh environmental conditions, and in some aspects it is considered an analog of Mars [25]. The GTS measurements were compared against standard and calibrated measurement devices.

In Figure 8a we can see the different components of the GTS Antarctic experiment. On the upper part of the mast, there is an air temperature sensor inside a solar radiation protective case (note that on Mars, REMS will also provide air temperature measurements), whilst the data acquisition system, including battery and solar panel, is located on the lower part of the mast. To validate the data products retrieved from the GTS measurements, the ground temperature was also monitored by means of two different standard instruments: a calibrated Kipp and Zonen CNR1 net radiometer or pyrgeometer (right part of the mast), and a contact temperature sensor based on a RTD, a Pt100, and located exactly in the middle of an aluminum square plate (10 × 10 × 0.5 cm). The CNR1 net radiometer, located at approximately 1.5 m high and facing the ground, is based on a thermopile, such as the GTS, and its measurement band expands from 5–50 μm. The contact sensor was buried in the ground, within the FOV of the GTS, and at approximately 1 cm below the surface.

The GTS Antarctic engineering model and its amplification electronics were located inside a box placed in a horizontal position on the left side of the mast (Figure 8a). The GTS faced the ground on the bottom of the box at an approximate height of 1.5 m above the ground, giving a FOV radius of approximately 0.86 m. The GTS engineering model used for the field test included a thermopile in the 8–14 μm band and the in-flight calibration plate. The readout electronics system did not use the REMS ASIC, and it was designed specifically for this application. The output voltage of the thermopile was amplified by a low noise and precision instrumentation amplifier AMP01 by Analog Devices, with a gain of 1,924. The amplifier output was filtered to limit the maximum output frequency to 40 Hz. The input to the amplifier was sampled for a period of 5 min, and it alternated between the thermopile output and a resistance of the same value for the thermopile impedance, which enabled us to measure the offset introduced by the amplifier. A Grant 4F16 datalogger was used for the sampling and recording system, which, in addition to the amplified thermopile signal, stores the internal resistance of the thermopile and calibration plate Pt1000 temperature sensors and the previously described auxiliary sensors used for the field tests.

### 4.1. Environmental Model to Determine Ground Soil Temperature

The values for Antarctic soil emissivities *ε_soils_* at the deployment location for the GTS and the CNR1 net radiometer band pass were measured [13] (Table 6). Since these values are lower than 1, a fraction of the radiation emitted by the atmosphere is reflected on the surface, and also on the detectors. This radiation must be taken into account and subtracted from the total heat flux signal, if the kinematic temperature of ground soil is to be measured. Equation (8) proposes a simple model for total incident heat flux in the thermopile:

(8) $$\Phi_{g}=\varepsilon_{\mathrm{soils}}\cdot \Phi_{\mathrm{soil}}+\left(1-\varepsilon_{\mathrm{soils}}\right)\cdot \varepsilon_{\mathrm{air}}\cdot \Phi_{\mathrm{air}}$$

where *ε_air_* is the effective emissivity of the atmosphere, the value of which is obtained following the models presented in [26] (Table 6).Φ*_air_* is the heat flux term of the atmosphere calculated using Planck’s law and the measured temperature of the air. Finally, to obtain the temperature of the ground soil, we need to solve Equation (5) for Φ*_g_* and from that value Equation (8) can be finally solved for Φ*_soil_* thus obtaining the value of ground soil kinematic temperature.

### 4.2. Results from the Antarctic Campaign

The main objective of the Antarctic field campaign was to validate the data products given by REMS GTS, compared with commercial and calibrated instruments, as well as to test the correct performance of the in-flight calibration procedure. Data have been processed with the model described in the previous section, whilst the constants of the engineering model used were calibrated previously, following the test set-up and procedures described in [24].

**Figure 9.**
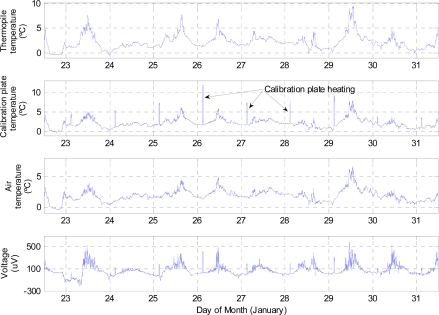
Output signals of the GTS engineering model during the measuring period.

**Figure 10.**
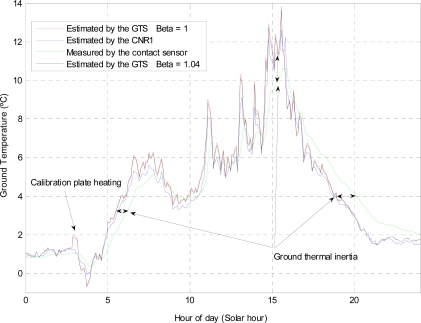
Ground temperature estimated by the GTS in comparison with calibrated CNR1 net radiometer and contact RTD sensor (Pt100) for the 25th of January.

Figure 9 shows the GTS electrical variables measured by the datalogger during the entire test campaign. In the graphs, it can be seen that the calibration plate was heated for 20 minutes every day before dawn (four sampling periods). Although the heating power of the calibration plate was constant, due to different weather conditions, mainly winds, the temperature of this plate only rose above 5 K on five occasions (note that Earth’s atmosphere is denser than that of Mars, increasing forced and natural convection and its cooling capacity). In Figure 9 can also be observed that the working temperatures of the GTS varied between 273 K and 283 K, while the air temperature changed between 273 K and 280 K. Using the data recorded for the 25th of January, Figure 10 shows the result of applying the GTS measurement procedure; assuming that the thermopile filter is clean, this is *β* = 1. Table 7 clearly illustrates that there is a very good agreement between CNR1 net radiometer and GTS data. However, diurnal discrepancy is greater because of GTS thermal gradients due to the effect of the sun and/or the wind. Nevertheless, there is a slightly larger difference between the GTS measurements and the data obtained by the contact sensor (Table 7). This difference may be caused by several different factors, including the depth at which this sensor was located. Both the GTS and the CNR1 net radiometer are based on infrared radiation coming from the surface, so they measure the skin temperature of the soil, *i.e*., the temperature of the first ground microns. However, the contact sensor, as stated previously, was located approximately 1cm below the surface, and thus measured the temperature at this depth. A further factor is that the temperature evolution of the contact sensor is smoother, denoting the higher thermal inertia of the ground. For example, the diurnal evolution of the GTS and the CNR1 net radiometer temperature estimations presents sudden changes created by the effect of the sun and/or the wind, which modify the skin ground temperature without affecting the deeper soil layers.

**Figure 11.**
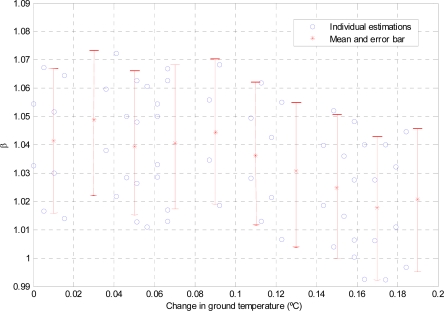
Specific and average values for estimation of *β* constant.

The in-flight calibration procedure was also tested during the Antarctic campaign. The differential algorithm was executed using data from the 26th of January, selecting fifteen samples taken before and four taken during the calibration plate heating process. Thus, with all possible combinations of samples (heated-unheated), data pairs for execution of the algorithm were created. Figure 11 shows the individual values of *β* for each selected pair versus the variation of ground temperature, and the mean and 3σ error bar for steps of 0.02 K of variation in ground temperature. The ground temperature variation was measured using the buried contact sensor. The individual values were contaminated by the measurement noise of electronics and the datalogger channels, to which the in-flight calibration algorithm is especially sensitive since it is the result of a quotient, Equation (7).

The average values for *β* varied between 1.05 and 1.02, which is slightly larger than the expected value of 1, which corresponds to a thermopile with a clean filter. It is very important to note that a change in ground temperature of 0.2 K during the execution of the in-flight calibration algorithm implies a modification of 3% in the estimated value of *β*. This is because the in-flight calibration algorithm is based on the assumption of a constant ground temperature, Equation (7). Thus, the in-flight calibration must be executed just at dawn, when surface temperature is expected to be more stable than during the rest of the day. Unfortunately, during Martian operations the evolution of *β* versus ground temperature drift will not be available since although a temporal evolution of the value of *β* will be known, ground temperature will remain unknown. Therefore, only if average values of *β* are stable will the calibration results be validated. In the present case, the average value of *β* for a null drift in ground temperature is 1.04, denoting a 4% of improvement in the gain or sensitivity of the entire system. This increment may be caused by a change in thermopile sensitivity due to the calibration thermal cycles, the relative movement of the calibration plate modifying slightly the value of the calibrated constant *α*, or even due to a change in the gain of the read out electronics. Figure 10 also shows the ground temperature estimation for the new value of *β* 1.04. Table 7 shows the slightly smaller RMSE values in comparison with the reference sensors, denoting the correct estimation of *β*.

## 5. Conclusions and Future Work

A detailed description of the design and modeling of a contactless ground temperature sensor for Mars has been given. The sensor is based on the use of three thermopiles, and the key parameters of the design are: (a) the sensor’s mechanical and electrical simplicity, based on the use of thermopiles which are robust when presented with temporal and spatial gradients, and require relatively simple conditioning; (b) the design of an in-flight calibration system with no moving parts, which rectifies sensor degradation due to dust deposition over the sensor surface or changes in thermopile sensitivity; (c) the use of a set of mathematical equations which have enabled us to model the physical operation of the entire sensor.

The GTS engineering model has been demonstrated to be capable of measuring the temperature of the ground under realistic test conditions, as well as having the capacity to calibrate the sensor during normal operation, using the proposed in-flight calibration algorithm. The performance of the sensor has been evaluated in comparison with a commercial CNR1 net radiometer showing a high degree of fitness, with an error below 0.5 K.

For future research, two tasks remain pending: firstly, an assessment of the capability of operating with the output signal of two thermopiles in order to apply color pyrometry techniques, estimating not only the temperature of the ground but also its emissivity, and avoiding the uncertainty associated with this value. And secondly, deployment of the instrument in a dusty environment to provide a better test of in-flight calibration system performance.

## Figures and Tables

**Table 1.** General methods to measure ground temperatures.

**Ground temperature measurement method**	**Advantages**	**Drawbacks**
Contact sensor	- Technically simple. - Gives the real kinematic temperature.	- Very localized measurement. - Gives the temperature where there sensor is buried, which may differ from the skin temperature (layer of dust). - Mission technical restrictions.
Contactless sensor	- Gives the skin temperature, *i.e.*, the temperature of the first microns of the surface. - Possible to measure over a large area, to avoid local effects in the temperature. - Possible to measure the temperatures of different points moving the sensor.	- Technically more complex. - Gives the brightness temperature of the surface. - Needs a correction from atmospheric effects. - The emissivity of the surface and the atmospheric emission are needed in order to give the kinematic temperature. - Atmosphere absorbance (transmission windows).
Contactless sensor with color pyrometry	The same as the standard contactless, technique but also: - Gives the real kinematic temperature.	- Needs at least two measuring bands and a very good estimation of atmospheric effects, so more complex than the standard contacless technique.

**Table 2.** GTS general characteristics (without electronics) and required performances.

**GTS Property**	**Value**
Dimensions	40 × 28 × 19 mm
Mass	20 g
Temperature working range (*T_C_*) (Min – Max)	150–300 K
Ground temperature range	*T_C_* ± 40 K
Resolution	0,1 K
Accuracy	±5 K
Field of view (FOV)	60°(horizontal), 40°(vertical)

**Table 3.** GTS sensors and actuator characteristics.

**GTS Item**	**Wavelength Sensitivity**	**Unit**	**Range Min-Max**	**Sensitivity Min-Max**
Thermopile A (Group of thermocouples)	8–14 μm (average transmittance 75%)	Volts	±1.6m V*	35–70 μV/K*
Thermopile B (Group of thermocouples)	15.5–19 μm (average transmittance 65%)	Volts	±0.64 mV*	12–16 μV/K*
Thermopile C (Group of thermocouples)	14.5–15.5 μm (average transmittance 65%)	Volts	±0.25 mV*	4.7–6.6 μV/K*
Pt1000s (Platinum thermo- resistor)	-	Ohms	450–1,100 Ω	3.85 Ω/K
Heater (Electrical heater)	-	Ohms	41 Ω	-

* These data depend on the specific thermopile and its working temperature.

**Table 4.** ASIC amplifier parameters.

**Parameters**	**Thermopile Channels**	**Pt1000 Channels**
Full Scale (Differential Output)	4 V_P-P_	4 V_P-P_
Nominal Bias Current	-	1,000 μA (62.5 ms)
Amp Gain	64, 128, 256, 2,048 V/V	4 V/V
Input Offset	5 μV	10 μV
Input Differential Impedance	3,000 MΩ	-
Amp RMS Noise Contribution	15 μV_RMS_/√Hz	15 μV_RMS_/√Hz
CMRR (DC)	66 dB	66 dB
Gain Absolute Accuracy	1%	-
Analog Chain Stability	-	0.1 K
Beginning-of-Life (Output Amp Accuracy)	-	0.15 K
End-of-Life (Output Amp Accuracy)	125 μV	0.2 K

**Table 5.** GTS data production and power budget.

**Operational Mode**	**bits/s**	**kbits/sol**	**Power(mW)**	**Energy/sol(mWh)**
Nominal (1sps during 5 min each hour)	112	806.4	0.25	0.5
In-flight calibration (1sps during 15 min each week)	112	14.4	500	17.8

**Total**	-	820.8	-	18.3

**Table 6.** Soil and atmosphere effective emissivities.

**IR detector**	**Soil emissivity (*ε_soils_*)**	Atmosphere effective emissivity (*ε_air_*)*
GTS	0.97	0.92
CNR1 net radiometer	0.96	1

* For cloudy conditions, those found on Antarctica during the test campaign.

**Table 7.** GTS compared with CNR1 net radiometer and contact sensor for the 25th of January data.

**RMSE (Root Mean Square Error) [K]**	**CNR1 Net Radiometer**	**Contact Sensor (Pt100)**
**GTS *β* = 1**	0.44 K	1.06 K
**GTS *β* = 1.04**	0.39 K	1.03 K
